# I-mindfulness-based cognitive therapy (i-MBCT) in the treatment of COVID-19 related adjustment disorder. a RCT study with active control group

**DOI:** 10.1192/j.eurpsy.2021.291

**Published:** 2021-08-13

**Authors:** P. Holas

**Affiliations:** Faculty Of Psychology, University of Warsaw, Warsaw, Poland

**Keywords:** Adjustment Disorder, mindfulness, COVID-19, MBCT

## Abstract

**Introduction:**

Adjustment disorder (AD) is described as a maladaptive reaction to an identifiable psychosocial stressor/s that usually emerges within a month after the onset of the stressor. With all uncertainty, fears and disorientation, it’s no surprise that many people have developed an AD linked to the sudden changes brought about by COVID-19, such as threat to life, imposed restrictions, and the associated changes. Mindfulness-based cognitive therapy (MBCT) has been found to be effective for depression and anxiety problems, little is known, however, about its efficacy for adjustment disorder.

**Objectives:**

The aim of the current research was to evaluate if 4 weeks long, modified internet-delivered MBCT can reduce symptoms of Covid-19 related AD.

**Methods:**

438 individuals with a diagnosis of AD were recruited to take part in the study. They were randomly assigned to i-MBCT, i-progressive muscle relaxation training (i-PMR), and Waiting List (WL). Assessments with questionnaires evaluating AD (ADMN-20), depression (PHQ-9, HADS-D), and anxiety (HADS-A, GAD-7) were filled at baseline, 4-week, and 1-month post-randomization. 142 individuals completed baseline and 4 week assessment (i-MBCT, n= 34; i-PMR, n= 36 and WL, n=72).

**Results:**

We found a significant reduction in AD symptoms following the i-MBCT group, whereas no change was found in both control conditions. While a decrease in depressive and anxiety was found in both i-MBCT and i-PMR groups, the greatest reduction has been observed in i-MBCT.

**Conclusions:**

These preliminary findings suggest that i-MBCT can be an effective intervention in treating Covid-19 related AD, but more studies are needed.

**Disclosure:**

No significant relationships.

